# Smart City + IoT Standardization Application Practice Model and Realization of Key Technologies

**DOI:** 10.1155/2022/8070939

**Published:** 2022-02-09

**Authors:** Chunqiao Song, Xutong Wu

**Affiliations:** ^1^Faculty of Innovation and Design, City University of Macau, Macau 999078, China; ^2^Macau University of Science and Technology, Institute of Social and Cultural Research, Macau 999078, China

## Abstract

Smart city gathers heterogeneous information, which requires a unified data access and collection mechanism. In this paper, based on the computing framework of IoT put forward to satisfy urban sensing network data acquisition, transmission, and computing technology system, according to the function requirements of IoT gateway, we put forward the method of building software system and hardware system and the urban perception network middleware architecture that can meet the demand of M2M, which can realize dynamic management of cognitive resources. It also supports ubiquitous services over HTTP. This is of great significance for providing intelligent services with diversity of solvers, controllers, and computing terminals. This paper focuses on how to comprehensively apply intelligent sensing, wireless transmission, data mining, and other technologies of the Internet of Things to associate data with typical application examples such as vehicle sensing and positioning technology and urban security sensor application. These typical applications can effectively solve the practical problems in urban operation and management, meet the needs of the expansion of the concept of Internet of things in urban management and the continuous deepening of management, and improve the scientific and intelligent level of urban operation and management.

## 1. Introduction

As the trading center and gathering center of human beings, city is the product. The emergence of cities is the symbol of human society entering the civilized era, and also the advanced form of human group life. The continuous acceleration of urbanization has led to the rapid increase of urban population, the rapid expansion of urban scale, and the continuous enhancement [1]. It improves the level of urban services and promotes the sustainable and leapfrog development of cities through comprehensive and transparent information perception, wide and safe information transmission, and intelligent and efficient information processing. In this way, a new form of urban development can be constructed, so that the city can automatically perceive and effectively make decisions and achieve control [2]. As the infrastructure of smart city, information technology is undoubtedly composed of the three most core technology hot spots of the Internet of Things, cloud computing, and data association technology. In fact, these three technologies all belong to the general description of the platform including many technical branches. Internet of Things technology [3] is based on Radio Frequency Identification RFID and other sensing devices.

From the perspective of information technology, “wisdom city” intelligent information technology in the city has “four” characteristics: ubiquity, utility, intelligence, and greening [4]. (1) Ubiquity: the application of information technology is everywhere, people and nature, man and society, and nature and society are linked in the pan in the network information for smooth circulation and fusion. The network has become the basic production tool of the information society [5]. (2) Utility: the deployment, application, and management of IT infrastructure will be the same as those of water, electricity, and gas, with the principle of “centralized service, on-demand use, virtual ownership and easy to use” [6]. (3) Intelligence of infrastructure and its applications is more convenient. Information sharing technology under data fusion and application system with intelligent perception and respect for user experience affect all aspects of people's lives. Smart city will shape the vision of a better life “benefiting everyone” [7]. (4) Flexibility: “ubiquitous information acquisition equipment; unimpeded information transmission channels; information sharing under security guarantee; powerful data processing centers; and intelligent software and services play more roles in urban operation than hardware production and manufacturing [8]. Concepts such as “smart Earth” and “smart city” have emerged. “Smart city” is a new urban form with learning, adaptation, and innovation capabilities. Based on the network integration of Internet of Things, telecommunication network, wireless network, and other networks, and using a variety of high-tech tools, it establishes an urban ecosystem including government, citizens, and commercial organizations [9]. It can be seen that the Internet of Things actually includes three entities: human, machine, and object. Intelligent devices are used to sense and acquire information about objects and their surroundings through recognition and perception technology [10], and then information and knowledge are transmitted through network transmission and communication technology and equipment. Finally, through the intelligent message retention, and knowledge processing technology and equipment to achieve intelligent management and control of objects or the physical world of a kind of “object perception, people interconnection, everyone interoperability” efficient, intelligent network, database and intelligent computing technology (including artificial neural network and evidence combination), standardization technology, etc. [11], literature [12] applies sensor data fusion technology to activity recognition in the field of smart home. Considering the advantages of intelligent computing, researchers gradually design and use intelligent algorithms to carry out data fusion research. For example, the improved particle swarm optimization algorithm is applied to fuse the multisensor information in the Internet of Things for precise measurement [13]. Based on the event-driven central point fusion algorithm, a fusion timing control mechanism based on differentiated services is proposed [14]. Cloud computing, comprehensive perception, intelligent computing analysis, ubiquitous connectivity, digitalization, information, and network of modern cities are new in recent years due to the integration and convergence [15]. The concept of “smart Earth” proposed by IBM elevated smart city construction into a national strategy [16]. Next, with wisdom city construction mainly aimed at people widespread concern in the construction of the city of urban traffic congestion, public security, network optimization deployment and nondestructive testing, digital city life, medical care, urban greening, and sudden disasters, etc. [17] make full use of the Internet of things technology in the intelligent sensing and comprehensive perception, information sharing, and service advantages, promote the improvement of modern social civilization and government management and service level, and comprehensively improve the happiness index of urban and rural residents [18, 19]. Through the construction of urban operation monitoring platform based on the Internet of things, realize the combination of automatic and manual new regulatory model, effectively improve the government guarantee public security and the ability to deal with emergencies, maximum limit to prevent or reduce damage [20, 21], safeguard the life property safety of the public, safeguarding national security and social stability, and promote social economic comprehensive, harmonious, and sustainable development. The combination of the Internet of things and urban management is getting closer and closer. At present, there are application solutions of the Internet of things technology in urban management, environmental monitoring, logistics informatization, intelligent transportation, public security, and many other fields [22, 23], and demonstration work has been actively carried out in all aspects of the city [24].

This article first analyzes the metropolitan area network perception function definition and hierarchy, and then according to the data acquisition and exchange demand of the city of wisdom, this paper proposes a IoT gateway hardware and software of the system, then considers wisdom city service framework based on cloud computing, and discusses the design principle of metropolitan area perception network middleware and implementation method of the system architecture. Finally, the above main architecture is verified by performance test experiments. Using the theory of innovation and technology to build a series of specific function models, this part will analyze the diverse needs of construction of urban management wisdom, using the IFC standard, data mining, and text mining method, combined with a variety of emerging information technologies to achieve the extraction of submodels, definition, and integration, such as the generation process; this is part of the model to study further on. It is also a key link between the past and the future.

## 2. Key Technology System Architecture of Smart City Internet of Things Application Practice Model

### 2.1. Application Practice Model of Intelligent City Internet of Things Research Technical Framework

The technical architecture of a typical Internet of Things system includes three parts: perception layer, transmission layer, and application layer. This training system adopts typical architecture design, and the functions of each layer are as follows:

#### 2.1.1. Perception Layer

Look for the training function, cultivating students perception layer function, the characteristics of a variety of sensors, detection methods, cognitive, and selection ability, for the application of single chip microcomputer (51 series and CC25xx series) programming ability, and the business requirements and the engineering application environment, according to the system planning of the node deployment method and the construction deployment according to specification.

#### 2.1.2. Transport Layer

From the perspective of system composition and technical functions, and from the perspective of practical training function, the transmission layer focuses on cultivating students' skills in the configuration and application of Internet of Things products, as well as the application development ability of SCM (CC25xx series) or embedded processor.

#### 2.1.3. Application Layer

**Figure 1:**
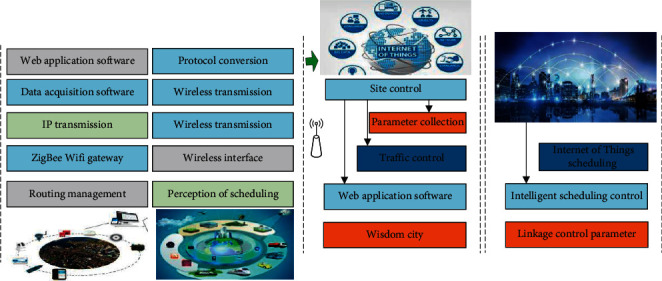
Block diagram of smart city + Internet of Things traffic scheduling control system.

The smart City + IoT traffic scheduling system is used to monitor commuting at important traffic crossings (such as Bridges) and control the operating status of traffic lights at corresponding locations. The system composition is shown in Figure 1. Subsystem operation and lighting control subsystem, the user through a Web application software, set up bridge state of overload, the linkage of the road traffic status, traffic lights to run the action control parameters, collection and service software issued instructions according to requirements of the linkage control and feedback data, through a Web application software system running state.

The concept of smart city IoT standardization is based on the premise of urban IoT standardization informatization and the development of emerging information technology in China. It is an effective means to realize the whole life cycle management of construction projects, which can promote the optimization of urban IoT standardization related professionals and personnel and change the content and mode of construction project management. Innovation and integration of a variety of emerging information technology are the core of wisdom city of standardization of the Internet of things, but the wisdom city IoT standardization idea advocated is not only a collection of a large number of systems integration and information infrastructure, but also the whole life cycle of construction project management and multiple parties together, creating wisdom, integrated with the construction of human environment, providing convenient, efficient, and efficient project management methods. In order to achieve these goals, the concept of smart city IoT standardization emphasizes the establishment of anthropomorphic sensing, connectivity, and intelligence functions, as well as harmonious coexistence with the construction environment to achieve sustainable development of construction projects. Based on the above analysis, the conceptual model of IoT standardization concept of smart city is obtained, and the causes, management objectives, main features, and realization mechanism of IOT standardization concept of smart city are revealed.

## 3. Internet of Things Contributes to the Refined Model and Key Technologies of Urban Management

**Figure 2:**
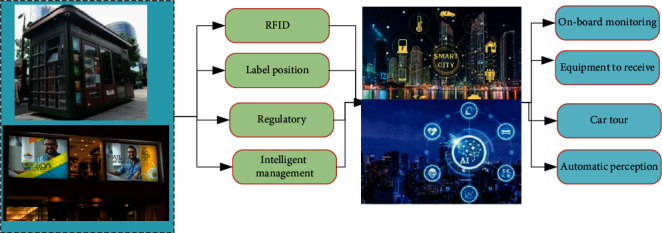
Schematic diagram of vehicle-mounted monitoring law enforcement methods.

Effective supervision of movable objects (breakfast carts, newsstands, etc.) and frequently changed objects (advertisements, plaques, etc., of shops on both sides of the street) can make full use of radio Frequency Identification (RFID) tag positioning technology, the core technology of the Internet of Things. The legal and compliant supervision objects shall be installed with identifiable electronic tags. The vehicle-mounted monitoring and law enforcement system is established based on the integrated supervision functions of base stations, panoramic cameras, and other equipment to realize vehicle-mounted patrol, automatic perception, real-time positioning, and rapid investigation and evidence collection of supervised objects and improve the efficiency and intelligence level of management. The supervision mode is shown in Figure 2.

With the continuous improvement of people's living standard, city parks, temple fairs, or the famous tourist attractions such as area in a particular time period (such as holidays or during certain activities frequently, people “blowout” phenomenon, it also makes the crowds gathered risk further ascension and increased security hidden danger, and the crowd safety management difficulty also will increase. Traditional management mainly adopts the way of on-site monitoring with a large number of manpower, but it can only play the role of alarm disposal and lacks the prediction and early warning of crowd gathering risk.

(1) $$\begin{array}{c} f_{p}=\min\sum_{d}\frac{D_{J}}{p}. \end{array}$$

1. In the multiobjective optimization model, both models have shortcomings in measuring progress in improving optimization. Multiobjective Selection Based on Dominated Hypervolume (SCPIMM) model showed better performance and optimized results for different front shapes.
2. According to the group based chain coding method, the parent individuals were initialized to generate a parent population of size N, and the fitness of individuals in the population was evaluated.
3. A rapid nondominant ranking was carried out on the evaluated parent population to classify the dominant rank of individuals in the population.
4. Population evolution operation: the algorithm adopts the tournament selection method of two parent individuals selected from the population, and to improve the two parent individuals type single point cross to generate a hybrid individuals, and then to intelligent hybrid individual variations, get a child individual, pair the last individual fitness evaluation, and add the individual child population pool. Repeat this process until the subpopulation size is N.
5. Optimal N individuals were selected as the next generation parent population by using hypervolume-based population renewal mechanism for parent population and child population.

## 4. Information Model Construction of Intelligent City Internet of Things

**Figure 3:**
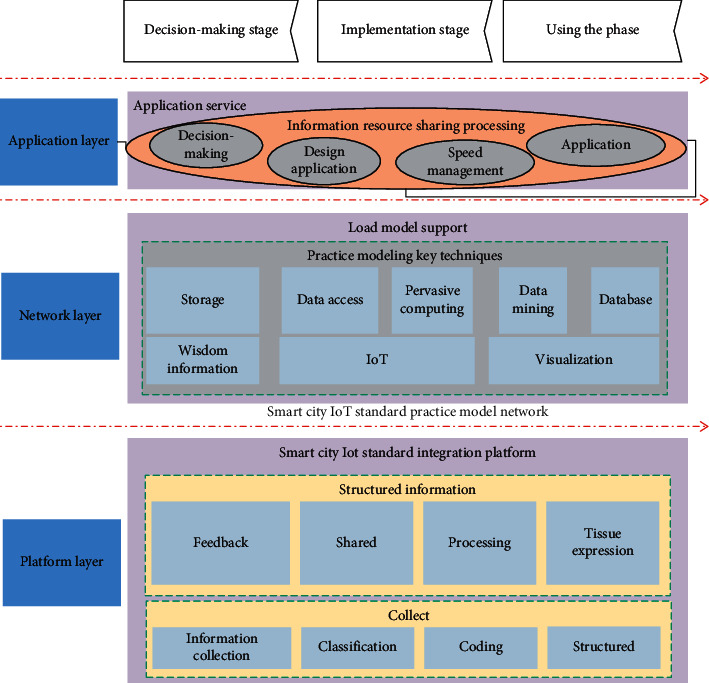
Main frame of IoT standardization information model for smart city.

The modeling process should reflect the main features of the standardization concept of the Intelligent city Internet of Things, integrate a variety of emerging information technologies into the model, and meet the needs of modern construction project information management. SCPIMM can not only meet the individual needs of different participants in the process of construction project management for information management, but also realize the effective management and control of the whole life cycle information and improve the efficiency and level of construction project information management. The basic framework structure of SCPIMM proposed in this paper is shown in Figure 3.

1. Data layer can be divided into structured, unstructured, and process information according to the structural characteristics of construction project information. For different information structures, SCPIMM has established three kinds of databases to realize classified storage and management: the storage and management of structured information are carried out through the structured information database constructed by the model. The unstructured information is stored and managed through the unstructured information database constructed by the model by establishing the link relationship between the model and the document. The process information of construction project is stored and managed by process information database according to the principle of mapping. According to the characteristics and practical experience of SCPIMM, the model database serves as the carrier and storage terminal of project information and takes information exchange interface and middleware technology as the key to process and process heterogeneous information, providing support for other levels of the model.
2. In the process of construction project information management, the platform layer uses sensors and information perception technology to build an information management platform with perceptive ability and real-time information interaction, so as to realize the collection and monitoring of construction project information resources and complete the information management work of the whole life cycle of the model. The platform layer of the model is composed of a variety of sensors, generally including HD camera, concentration sensor, temperature and humidity sensor, two-dimensional code label, radio frequency label reader, GPS, and other sensing terminal devices. According to different stages and applications of the whole life cycle of construction, the platform layer can classify and encode all the collected information and process the information according to structured, unstructured, and process information classification.
3. The network layer of the network layer model can obtain and process the information provided by the platform layer and build a network system by utilizing high-performance computing, data mining, visual management, and other functions provided by emerging information technologies such as the Internet of Things, ubiquitous computing, and 4D visualization. Its core capability is the transmission and processing of information. Information network construction, based on the technology of Internet of things, can be achieved from a single project to the whole construction project information management of specific equipment, the whole process of information interaction, from the construction project detailed information to the terminal transmission path through the network, sensor, information storage database, to the network infrastructure, realizing information sharing and interaction.
4. The application layer of the application layer model uses a variety of emerging information technologies to form a whole-process intelligent information management process from event prediction to decision assistance and emergency linkage and to create a comprehensive management system of information collection, classification and coding, organization and expression, processing and processing and transmission, sharing, and feedback. The application layer of the model is composed of application software with different functions, including investment analysis, architectural design, structural design, cost management, schedule control, quality control, safety management, and energy consumption analysis. The application layer of the model is the interface between the network layer and the user, which combines with the user's needs and adopts P2P access mechanism based on the network topology structure to provide different services for different participants.

The design of information extraction mechanism is the premise of submodel construction, which is directly related to the efficiency of information management of construction projects. The main purpose of information extraction is to obtain the information sample similar to the information needed at present. In this paper, the submodel information extraction mechanism is designed according to the similarity principle. The following three methods are adopted to define the similarity between the target and the sample in the extraction process.

(1) Similarity between information is defined by Euclidean distance P.
  (2) $$\begin{array}{c} d_{p}=\sum_{i}W_{i}{\left(p_{\text{ok}}-p_{i}\right)}^{2}. \end{array}$$
(2) Similarity of information is defined by Manhattan distance.
  (3) $$\begin{array}{c} d_{p}=\sum_{p}W_{p}\left|p_{\text{ik}}-p_{i}\right|. \end{array}$$
(3) Similarity between information is defined by infinite modulus distance.

(4) $$\begin{array}{c} d_{p}=\max W_{i}\left|p_{\text{ik}}-p_{i}\right|. \end{array}$$

In the information preparation stage of the submodel, massive construction project information should be preprocessed by stem extraction (Stemming, stem segmentation, stop words removed, punctuation deleted, etc.). For English, stem should be extracted, and nouns, adjectives, conjunctions, and adverbs should be taken as feature items. For Chinese stem segmentation, namely, word segmentation, specific word segmentation techniques include maximum matching, reverse maximum matching, and traversal one by one. The feature items after word segmentation should be processed by dimensionality reduction, that is, selecting the required feature items f to form a set N.

(5) $$\begin{array}{c} p\left(d,t\right)=\frac{\mathrm{lg}\left(d,f\right)\left(N/n_{j}\right)}{\sqrt{\sum {\left(d,t\right)}^{2}\mathrm{lg}{\left(N/n\right)}^{2}}}. \end{array}$$

The application layer of associated data is mainly composed of Resource Description Framework (RDF) triplet evaluation, data visualization, semantic query and browsing, personalized information push, and other modules. First, the associated data automatically generated by the system is input to the application layer from the data storage and index layer. In RDF triplet evaluation module, experts and users score automatically generated relational data. The associated data retained after evaluation is displayed in the data visualization module through API isoform. For semantic query, browsing and other requirements in the application, semantic query, and Tabulator plug-in in browsing module can be realized. In addition, the personalized information push module is responsible for pushing the content that users may be interested in according to customized information.

## 5. Results and Analysis

**Figure 4:**
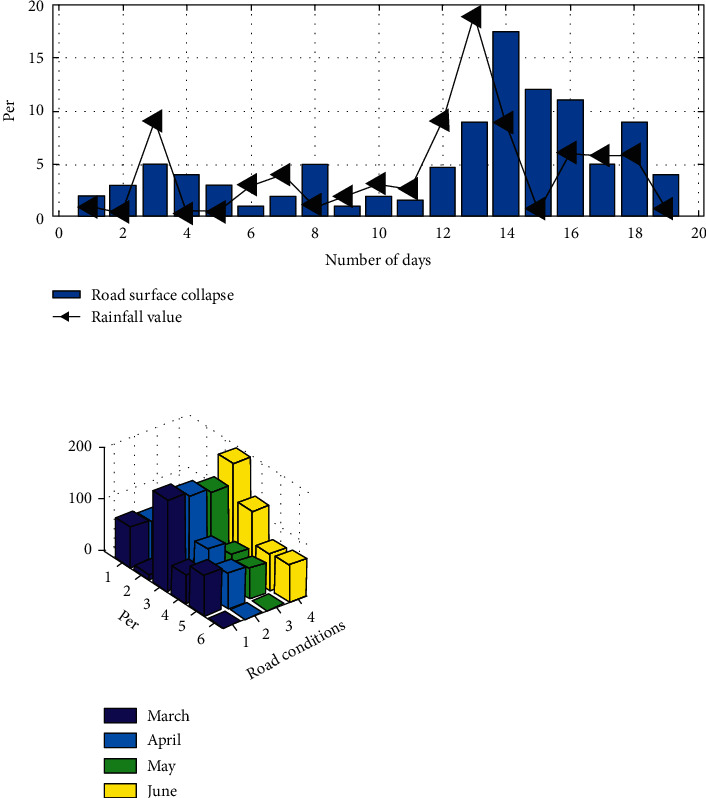
Correlation analysis of intelligent city IoT rainfall monitoring data and urban management case data.

The historical monitoring data accumulated by thousands of sensors in cities during the civil period can be an important part of the big data of flood control monitoring. On the basis of full investigation and investigation, special analysis of monitoring data and related data of other cities in the same period was carried out to find the consistency, concurrency, and derivative relationships among them, find the spatial distribution rules, and put forward scientific and reliable suggestions to better support flood control and emergency response work. For example, based on rainfall monitoring data, corresponding flood control risk assessment and early warning models can be established, and data mining and other means can be used to further improve the ability to master the law between rainfall and urban management cases, so as to realize the refinement of daily urban management related to flood control.  Figure 4 shows the analysis results of significant correlation between rainfall monitoring data and some types of management cases.

**Figure 5:**
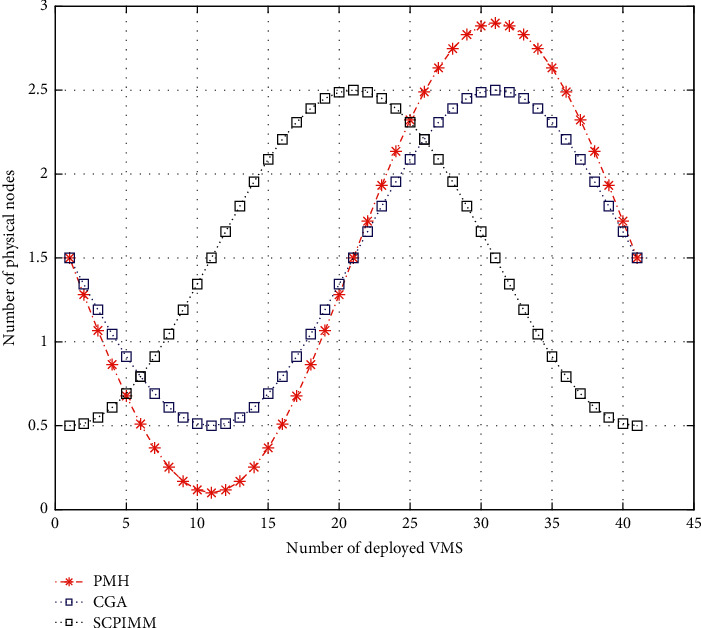
Schematic diagram of enabling physical node number.

**Figure 6:**
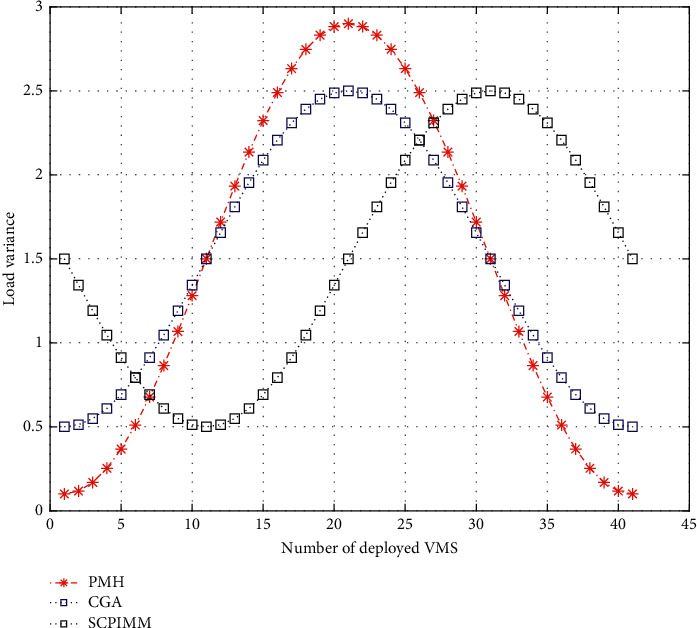
Schematic diagram of cluster load variance.

In terms of enabling the number of physical nodes, the number of physical nodes occupied by SCPIMM-based VM (Virtual Machine) deployment algorithm is almost the same as that occupied by CGA-based VM deployment algorithm and is smaller than that occupied by the priority matching heuristic (PMH) deployment algorithm, physical nodes. The smaller the number of tablets, the more the energy saving. In terms of the overall cluster load performance, the overall cluster load variances of the SCPIMM-based VM deployment algorithm are smaller than those of the two VM deployment algorithms. The smaller the load variances are, the better the load balancing effect of the server cluster is. Based on the above analysis; see Figures 5 and 6.

**Figure 7:**
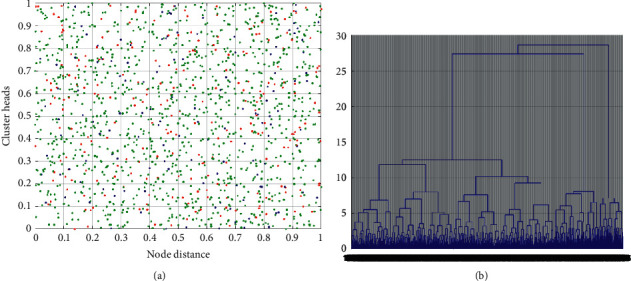
(a) Clustering formed by LEACH. (b) Clustering tree formed by SCPIMM.

To compare the network performance of the Structure-Conduct-Performance Interacting Multiple Model (SCPIMM) protocol with that of Low Energy Adaptive Clustering Hierarchy (LEACH) and SCPIMM, we first compare the clustering formed by the three protocols, as shown in Figure 7. It can be seen from the figure that the number of clusters formed by LEACH is too large, which is caused by the randomness of the selection of cluster heads. In LEACH, each sensor node decides whether to become a cluster head according to a certain probability, which has a strong randomness. The clustering formed by SCPIMM is better than that formed by LEACH, but it is different in size compared with SCPIMM. This is because the SCPIMM give full consideration to the state of the network to determine the optimal number of cluster heads, and then according to the residual energy of nodes, the distance between nodes and the distance between nodes and base stations to determine the optimal selection of cluster heads, thus forming clumps and uniform distribution, keep the optimal number of cluster heads, reduce the energy consumption of network, and improve the performance of the system.

**Figure 8:**
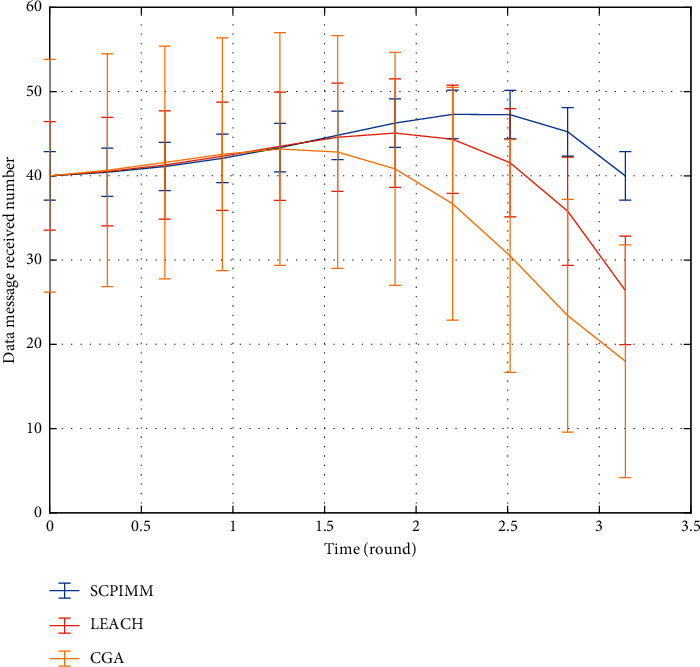
Data received by the base station.

The data received by the base station (sink node) is shown in Figure 8. We can see from the table that the proposed SCPIMM can receive more data, because we consider the residual energy of nodes when clustering, within the cluster distance and the distance with the base station, effectively reducing the energy consumption and reducing the amount of data transmission delay and improving the efficiency of data transmission, to send more data to base station. However, LEACH does not consider factors such as the remaining energy of nodes when clustering and randomly selects nodes as cluster heads. It is possible to select nodes whose energy is about to run out as cluster heads, resulting in data loss. Although SCPIMM considers residual energy and other factors when clustering, the optimal number of cluster heads does not change with the network state, and the influence of the distance between cluster heads and base station is not taken into account.

Based on the overall construction mode of smart city informatization service system, integrated Internet of Things construction mode, cloud computing construction mode, and intelligent infrastructure construction mode, and combined with the application demand of smart city people in smart city construction, the construction mode of smart city is explored and analyzed.

**Figure 9:**
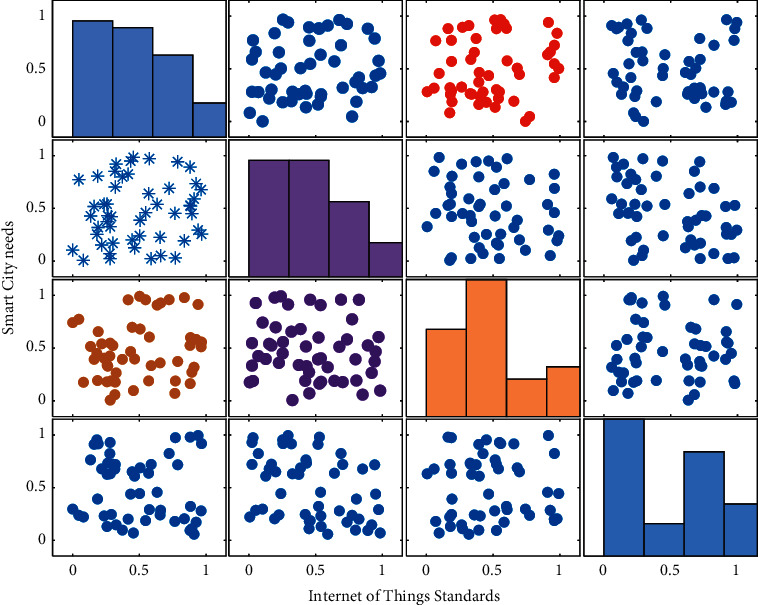
Application requirements of smart city construction.

According to the effective data collected from the questionnaire, overall, smart city citizens have higher demands for smart government, smart environment, smart medical treatment, smart transportation, and smart family in smart city construction, while their demands for smart energy and smart logistics are relatively low. See Figure 9.

**Figure 10:**
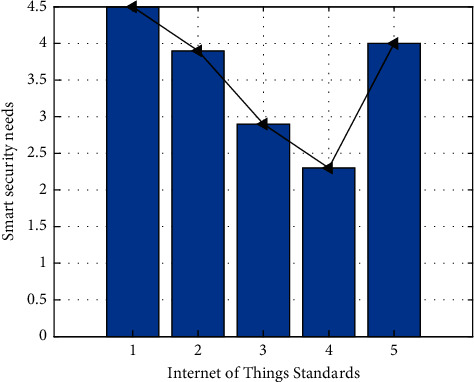
Intelligent security function requirements.

At the same time, from the point of view of intelligence security data, security is also a matter of great concern to the public, from the point of view of data, and the average score of security demand is 3.36 points. Among them, citizens' demands for early warning of natural disasters and emergency response of major emergencies account for a large proportion, accounting for 21% and 20%, respectively, as shown in Figure 10. The reason may be that the disasters such as earthquake, tsunami, and fire that have occurred in recent years are more serious, which make citizens have a kind of panic in psychology. They hope to take corresponding measures in the early warning of natural disasters and emergency response in case of major emergencies to avoid heavy costs. However, they did not pay much attention to the information tracking of registered population and permanent resident population, indicating that the citizens of smart cities can accommodate floating population, and the informatization of population and family planning in smart cities is also relatively good. Covering the food and drug traceability system, citizens are more concerned with the quality of the food and drug problems frequently recently, and also it is also a concern that citizens using the sensor monitoring food and drug trace the source of wisdom city that can be in shopping malls and supermarkets to install HD probe for video surveillance, clearly photographing food stand in front of the person's face, to prevent the poisoning of food and drugs.

The physical equipment layer is the infrastructure of a smart city, including access control devices, attendance devices, consumer machines, parking gates, and wireless sensor networks for sensing environmental changes. All devices access the information system of the data center through the Internet of Things gateway and communicate with the device abstraction layer of the intelligent building information system middleware. Middleware includes common service layer, sensor network management layer and application service layer. The sensor NMS topology consists of configuration services and abstraction layers. Topology and configuration services mainly realize the correspondence between physical space location of physical devices and information space location, and the interface of abstraction layer realizes the standardization of physical device access. The common service layer contains logging services, database services, and security services. The application service layer provides HTTP-based Web interfaces and local API interfaces for various applications.

## 6. Conclusion

At present, the urban management mode has undergone the transformation from digitalization and grid to urban operation, and its management direction is facing the development from digitalization to intelligence. These higher requirements for urban management and service work make the urban management must be based on the requirement of the people's fundamental starting point, adhering to the people-oriented, service for the people, to keep pace, through technology and management innovation to promote urban management work to a new level, to facilitate better break through the limitations of time and place, so that the people feel the role of urban management, enjoying the achievements of urban management. As an emerging technology integration means, Internet of Things technology can be applied in monitoring, transmission, analysis, and application of urban operation management information due to its characteristics of “intelligent identification, positioning, tracking, monitoring, and management” and has gradually become a bridge between “digital city” and “smart city.” It will be the ultimate goal and strategic direction of urban informatization to establish an intelligent and refined management mode of the city. As a breakthrough of the bottleneck of sustainable development of the city, it will bring a new look of the city in the future and promote the harmonious development of the city. In the next step, the Internet of Things application middleware needs to improve the design of loose integration with the upper application, adapt to sensor equipment more widely, and unify data standards; the improved data compression algorithm needs to be further improved in the actual environment and system.

## Data Availability

The data used to support the findings of this study are available from the corresponding author upon request.
